# Food consumption patterns in the Waterloo Region, Ontario, Canada: a cross-sectional telephone survey

**DOI:** 10.1186/1471-2458-8-370

**Published:** 2008-10-24

**Authors:** Andrea Nesbitt, Shannon Majowicz, Rita Finley, Frank Pollari, Katarina Pintar, Barbara Marshall, Angela Cook, Jan Sargeant, Jeff Wilson, Carl Ribble, Lewinda Knowles

**Affiliations:** 1 Department of Population Medicine, Ontario Veterinary College, University of Guelph, Guelph, Ontario, Canada; 2 Centre for Food-borne, Environmental and Zoonotic Infectious Diseases, Public Health Agency of Canada, 120-255 Woodlawn Road West, Guelph, Ontario, Canada; 3 Laboratory for Foodborne Zoonoses, Public Health Agency of Canada, 120-255 Woodlawn Road West, Guelph, Ontario, Canada; 4 Centre for Public Health and Zoonoses, & Department of Population Medicine, CLRE 203, Ontario Veterinary College, University of Guelph, Guelph, Ontario, Canada; 5 Faculty of Veterinary Medicine, University of Calgary, G380, 3330 Hospital Dr. N.W., Calgary, Alberta, Canada; 6 Region of Waterloo Public Health, Region of Waterloo, 99 Regina Street South, 3rd Floor, Waterloo, Ontario, N2J 4V3, Canada

## Abstract

**Background:**

The demographics and lifestyles of Canadians are changing, thereby influencing food choices and food preparation in the home. Although different dietary practices are associated with increased risk of foodborne illness, our ability to evaluate food consumption trends and assess risks associated with foodborne illness is limited by lack of data on current eating habits and consumer food safety practices. The objective of this study was to describe, for the first time, the food consumption patterns in a Canadian-based population from a food safety perspective, in order to establish baseline data on actual food intake of individuals.

**Method:**

A cross-sectional telephone survey of 2,332 randomly selected residents of Waterloo Region, Ontario, Canada (C-EnterNet pilot site) was conducted between November 2005 and March 2006. Food intake was assessed using a 7-day dietary recall method.

**Results:**

Certain food items were consumed more than others among the same food groups, and consumption of many food items varied by gender and age. Specific foods considered high-risk for the transmission of certain enteric pathogens were significantly more likely to be consumed by males (i.e. unpasteurized juice, bean sprouts, and undercooked meat) and elderly individuals (i.e. undercooked eggs). The majority of households prepared and consumed most meals at home, allocating an average of 44 minutes to prepare a meal.

**Conclusion:**

Baseline data on actual food intake is useful to public health professionals and food safety risk assessors for developing communication messages to consumers and in foodborne outbreak investigations.

## Background

Factors that influence food consumption choices among individuals and populations include cultural, social, and economic factors [1]. As food consumption patterns change over time, public health authorities and the food industry need to monitor the dietary intakes of the population [2].

In Canada, several sources provide information on food consumption patterns and nutrient intake. Food disappearance data and household food expenditure data have been used to identify national trends in food availability, and illustrate the dynamics of the food supply and consumer demands [3,4]. However, these data have limitations in that they do not measure individual consumption [5-7].

Individual dietary habits were first assessed by the Nutrition Canada Survey in 1970, providing valuable data on the intakes of several nutrients and the food consumption patterns of various sub-populations within Canada [8]. Current data on Canadian food consumption is limited to nutritional intake and status [9-11], or to specific subgroups such as adults [12,13], native communities [14], specific cultural communities [15], or rural populations [16].

Although it is important to monitor the nutritional status of the population, it is equally important to establish baseline data on actual food intake and practices of individuals. To address this, a survey on food consumption patterns from a food safety perspective was initiated in a Canadian community. The objectives of the study were to evaluate the patterns of food consumption in the general population, and to describe demographic factors that relate to the consumption of specific food items.

## Methods

This study was part of a broader cross-sectional telephone survey addressing food consumption patterns, knowledge of food safety principles, the prevalence of unsafe food-handling practices, and the prevalence of gastrointestinal symptoms, which was conducted in the Waterloo Region (Ontario, Canada) between November 2005 and March 2006 [17]. The Waterloo Region is located in southern Ontario, Canada, and is composed of three urban and four rural municipalities. Waterloo Region was selected since (a) it is the pilot sentinel site for C-EnterNet, the Public Health Agency of Canada's national integrated enteric pathogen surveillance program [18], (b) it includes both rural and urban settings, and (c) it has a population size of over 450,000, with diverse ethno-cultural communities.

The expected frequency of specific food consumption and handling behaviours deemed to be of most importance relating to the objectives of the study were used to determine sample size. We assumed expected frequencies of 24.3%, 32.2%, and 51.0% for eating egg dishes with runny yolk, using treated tap water, and washing hands after handling raw meat, respectively, in accordance with previous studies [19,20]. In addition, we considered an estimated prevalence of gastrointestinal illness of 10% per month [21], since a secondary objective of the overall survey was to evaluate the relationship between food consumption and symptoms of gastrointestinal illness. The final sample size of 2,300 was based on a combination of these calculations, using an allowable error of 2% and a 95% confidence. Sample size calculations were performed using Epi-Info 2004 Version 3.3 (CDC, Atlanta).

A multistage random-sampling design was used, and the seven municipalities within the study area were sampled proportionally as per the 2001 Census [22]. Households were randomly sampled from a list of residential telephone numbers (SelectPhone Canada™, InfoUSA Inc., version 5.2). The individual in the household with the next birthday was selected to participate in the survey provided they consented. If consent was not provided, the interview was terminated and the call status was recorded as not willing to participate. Respondents were considered eligible if they spoke English, were over the age of 18 months, and resided at a listed telephone number in the study area. Individuals were excluded from the survey if they had traveled outside of Canada within seven days prior to the interview, in order to capture Canada-specific food consumption.

Residential telephone numbers were attempted three times, with each attempt on different days and at different times of day. Each call attempt allowed a minimum of five rings. Once an eligible individual at a given household was identified, five attempts were made to contact that person to complete the survey. Any call-backs were scheduled on the day and time requested by the respondent. Proxy respondents were used, in particular, for participants less than 12 years of age who were less likely to recall their dietary intake over the past 7 days and for those between 12 and 17 years of age whose parent or guardian felt the child would not be able to answer the questions themselves. Proxy respondents were not accepted for individuals over the age of 18.

The research protocol was approved by the Human Subjects Committee of the University of Guelph (Guelph, Ontario, Canada). Informed consent was obtained verbally from all participants and from a parent or guardian of all respondents under the age of 18 years.

Interviews were conducted by trained interviewers at the Centre for Evaluation of Medicines (St. Joseph's Hospital, Hamilton, Ontario, Canada) using a computer-assisted telephone interviewing (CATI) system. The project manager monitored the first few interviews performed by each interviewer to ensure standardization of the survey tool. An Interviewer Manual was also provided to assist the interviewers with frequently asked questions and key definitions.

The survey was developed by modifying questions from existing questionnaires [23,24], with certain questions kept identical to facilitate future inter-survey comparisons. The survey covered 12 topic areas: (i) number of meals and dining locations, (ii) fruits and vegetables, (iii) dairy and eggs, (iv) alternative protein sources, (v) meats and seafood, (vi) water, (vii) home hygiene and food safety practices and knowledge, (viii) health (gastrointestinal illness in both one week and four weeks prior to the interview), (ix) demographics (age, gender, income, education, ethnicity, place of residence, pet ownership, number of children in household and household size), (x) grocery shopping practices and purchase of retail meat, including beef, chicken and pork, (xi) convenience foods, and (xii) food preparation, including meal preparation time and food handling knowledge and practice. All questions on food consumption addressed the respondent's consumption in the seven days prior to the interview. The interviewer read a list of food items to participants, and respondents were also given the opportunity to name food items they had consumed that were not on the list. Where appropriate, questions covering grocery shopping practices were asked of persons who were most familiar with these practices in the household, who may or may not have been the selected respondent.

The questionnaire was pre-tested in a small convenience sample of 6 individuals before applying it to the study population in order to identify questions that were confusing, ambiguous, or misleading, and to estimate the time required to complete the survey (average of 25 minutes). Data were analyzed using Intercooled Stata 9.1 for Windows (StataCorp LP, College Station, TX). Individuals who responded "don't know/not sure" or who refused to answer a question were excluded from analysis for that question. The Waterloo Region's population from the 2001 Canadian census was used to calculate expected population characteristics [22].

Prevalences were estimated for the consumption of all food items. Single food items that had less than ten responses were grouped into an 'Other' category for that specific food group. Exact confidence intervals (CI's) were computed for all proportions at the 95% level. These data were weighted to the 2001 Canadian Census by gender. Differences between gender and each food item consumed were tested using the Pearson's chi-square (χ^2^) test, and Fisher's exact test when the expected frequencies for a contingency table were less than five [25]. Age was categorized into six groups: ≤12 years of age (children), between 13 and 17 years of age (adolescents), between 18 and 24 years of age (young adults), between 25 and 54 years of age (adults), between 55 and 64 years of age (older adults), and ≥65 years of age (elderly). Pearson's χ^2 ^test or Fisher's exact test were also used to determine any differences between the age categories and each food item consumed. Statistically significant differences were determined by a two-tailed probability of less than 0.05. Odds ratios (OR's) were used to estimate the strength of association between food consumption patterns and demographic variables (adjusted for age and gender), where males and individuals 65 years of age or older were chosen as the reference groups. Although a large number of food items were included in this survey, we did not adjust for multiple testing. As a result, estimates are likely to have an increased type I error rate and fewer significant differences may exist than reported here.

## Results

### Study population characteristics

A total of 2,332 of the 7,142 contacted and eligible persons consented to be interviewed, yielding a response rate of 32.7%. A total of 87 ineligible individuals were unable to participate because they had traveled outside of Canada in the past seven days. Respondent characteristics are shown in Table 1. Compared to residents of the study area, respondents were older than the Census population, were more likely to be female, had a higher education level, and had a higher total annual household income.

**Table 1:** Demographic comparison showing percent of residents and survey respondents per category (except where noted) in the Waterloo Region, Ontario, Canada, November 2005 – March 2006

	Waterloo Region Residents (n = 438,515)	Survey Respondents (n = 2,332)
**Sex**		
Male	49.2	35.6*
Female	50.8	64.4*
**Age (years)**		
Mean	---	41.4
Median	35.3	40.0
0–12	17.9	7.7*
13–17	7.2	6.4
18–24	9.8	8.8
25–54	45.5	48.3*
55–64	8.4	13.0*
65+	11.3	15.8*
**Cultural group**^†^		
North American	---	65.1
European	---	11.4
African	---	0.2
Mediterranean	---	0.2
Asian	---	5.7
Native North American/Aboriginal	---	0.2
South American	---	0.8
Austral-Asian	---	0.04
**Education**		
No high school diploma	27.0	27.8
High school diploma	14.8	14.1
Some college or university	18.3	21.0*
College or trade diploma	28.0	14.1*
University, graduate, or professional	18.4	23.1*
**Total household income**		
<$20,000	13.6	9.9*
>$20,000 to <$40,000	20.4	17.0*
>$40,000 to <$60,000	19.8	20.3
>$60,000 to <$80,000	17.3	16.5
>$80,000 to <$100,000	12.0	15.8*
>$100,000	17.0	20.5*
**Residence**^‡^		
City or urban area	---	72.9
Suburban area	---	13.8
Town or village	---	9.1
Rural area	---	4.3
**Mean household size**	2.7	2.9
**Median household size**	---	3.0
**Mean # children in household**	---	0.9

* Proportion of respondents significantly different than the Waterloo Region residents, *P *< 0.05

^† ^Survey respondents reported with which cultural group they most identified. No comparable information was available from the 2001 Canadian Census.

^‡ ^No comparable information was available from the 2001 Canadian Census.

### Food consumption

The total proportions of each food item consumed by the study population during the seven days prior to the interview are presented in additional file 1. Of the 100% of respondents consuming produce, approximately 17% indicated that some or all of the produce they consumed was organically grown. Of the 55% of respondents eating nuts, 36% indicated that some or all of the nuts they had eaten were consumed raw. Of the 6% of respondents consuming tofu, 18% were vegetarian. Differences in consumption of food items by gender (adjusted for age) and age (adjusted for gender) are presented in Table 2 and in additional file 2, respectively, showing only those food items whose differences were statistically significant at *P *≤ 0.05. The proportion of respondents who reported that their food consumption patterns were typical of a normal week's food consumption are presented, by food category, in Table 3.

**Table 2:** Odds of a female respondent consuming a specific food item compared to a male respondent (adjusted for age), showing only those food items whose differences were statistically significant (*P *≤ 0.05) in the Waterloo Region, Ontario, Canada, November 2005 – March 2006

**Food Items**	**OR**	**95% CI^†^**
**(A) Herbs & Sprouts**		
Bean sprouts	0.75	0.56 < OR < 1.00

**(B) Fruits**		
Lemon	6.53	1.62 < OR < 26.26
Grapefruit	2.08	1.39 < OR < 3.11
Kiwi	1.73	1.24 < OR < 2.44
Raspberries	1.66	1.16 < OR < 2.38
Blueberries	1.58	1.21 < OR < 2.06
Pears	1.47	1.13 < OR < 1.91
Strawberries	1.36	1.12 < OR < 1.64
Grapes	1.23	1.03 < OR < 1.47
Unpasteurized Juice	0.70	0.50 < OR < 1.00

**(C) Vegetables**		
Beans	2.64	1.61 < OR < 4.33
Cauliflower	1.94	1.49 < OR < 2.54
Peppers	1.85	1.50 < OR < 2.27
Fresh celery	1.55	1.30 < OR < 1.85
Fresh carrots	1.43	1.15 < OR <1.79
Fresh spinach	1.42	1.13 < OR < 1.78
Broccoli	1.38	1.16 < OR < 1.65
Lettuce	1.33	1.04 < OR < 1.70
Other bulb onion	1.31	1.10 < OR < 1.57
Cucumber	1.22	1.03 < OR < 1.46
**Deli Salads**		
Pre-bagged, mixed salad greens	1.25	1.04 < OR < 1.50
Pasta salad	1.18	1.18 < OR < 2.04

**(D) Dairy**		
Yogurt	1.76	1.48 < OR < 2.10
White milk	1.43	1.10 < OR < 1.84
Sour cream	1.33	1.10 < OR < 1.61

**(E) Cheese**		
Gouda	2.28	1.18 < OR < 4.41
Cream cheese	1.66	1.37 < OR < 2.03
Cottage cheese	1.54	1.20 < OR < 1.97
Marble cheese	1.37	1.13 < OR < 1.68
Feta cheese	1.36	1.06 < OR < 1.75
Parmesan cheese	1.29	1.08 < OR < 1.54

**(F) Nuts**		
Almonds	1.27	1.04 < OR < 1.55
Pecan	2.14	1.28 < OR < 3.59

**(G) Meats**		
Ham	4.46	1.53 < OR < 12.81
Ground beef	1.34	1.13 < OR < 1.60
Steak	0.68	0.55 < OR < 0.82
Hamburgers	0.68	0.55 < OR < 0.83
Hamburgers not made in the home	0.60	0.47 < OR < 0.77
Hamburgers from pre-made uncooked patties	0.59	0.38 < OR < 0.93
Lamb	0.56	0.37 < OR < 0.85
Pink or undercooked pork*	0.45	0.24 < OR < 0.84

CI – confidence interval.		

*Among respondents that reported eating pork

^†^Exact 95% confidence intervals

**Table 3:** Percentage of respondents who noted that their reported food consumption patterns, as determined by this survey, were typical of a normal weeks' consumption, Waterloo Region, Ontario, Canada, November 2005 – March 2006

**Food Group**	**Typical of a Normal Week's Consumption**
	(%)
Fruits and vegetables	95.19
Dairy and egg products	95.50
Meats	91.98
Seafood	80.28

### Household dietary habits

In the seven days prior to the interview, respondents reported eating approximately 5.5 evening meals together with the majority of the household members, and spent on average a total of 44 minutes preparing a typical meal within the home. The study population reported eating roughly 4.3 meals or snacks per day. Respondents consumed approximately 2.4 meals or snacks consisting of leftover food items in the past seven days. Among respondents who ate leftover foods, 74% consumed the food within one to two days of initial preparation, 22% within three to four days, and 3% beyond four days. The number of meals in the past seven days, by dining location, is presented in Table 4. During an average week, respondents shopped for groceries 1.3 times. On average, 26% of food items purchased were ready-to-eat foods, 19% of food items purchased were ready-to-cook foods, and 50% of food items were purchased as basic raw ingredients.

**Table 4:** Mean, minimum and maximum number of meals prepared in the past seven days, by dining location, as reported by respondents in the Waterloo Region, Ontario, Canada, November 2005 – March 2006

Meal Location	Mean	Std. Dev.	Min.	Max.
Home	25.79	8.79	0	60
Outside home	3.34	3.83	0	35
Fast-food chain restaurant	0.69	1.43	0	20
Sit-down restaurant	0.56	0.96	0	9
Cafeteria or restaurant with buffet	0.38	1.20	0	15
Catered event	0.11	0.40	0	7
Pizza parlour or coffee/donut shop or street vender	1.20	2.15	0	21
Market deli or salad bar or ready-to-eat food from supermarket	0.31	0.83	0	14
Other restaurant	0.14	0.66	0	18

Around 12% of respondents followed a special dietary practice, of which approximately 5% followed special diets for various medical conditions, including those prescribed for diabetes. Vegetarianism was reported by 3% of respondents, while another 3% of respondents reported following special weight loss diets, and less than 1% reported following weight gain diets. Religious dietary practices or fasting was reported by less than 1% of respondents.

## Discussion

Previous food consumption surveys conducted in Canada have primarily focused on dietary intake from a nutritional perspective, or have used methods that provide little information on actual food consumption patterns. This study describes the food consumption patterns in a Canadian-based population, from a food safety perspective, presenting baseline data on actual food intake of individuals. The results illustrate that certain food items were consumed more than others among the same food groups, and that consumption of many food items varied by gender and age. The findings also demonstrate high levels of at-home food preparation and consumption.

### Food items

The extensive range of food items reported by survey respondents illustrates the food preferences of the population, and corresponds to the diversity of the Canadian food supply, as evidenced by food disappearance data [4] and the abundance of imported products, and foreign produce available throughout the year in grocery stores [11].

Within each food group, certain food items were consumed more than other items from the same food groups. The observed higher consumption prevalences of certain food items among the food groups are consistent with estimates of food availability within the Canadian food supply and consumer demands [4]. Comparing food availability data to the reported intake of food in populations is difficult since they represent different levels of dietary information [26], however, potential relationships may be identified.

Data depicting the amount of food available for consumption in Canada over the past two years [4] reveal that bananas, apples, white milk, cheddar cheese, eggs, chicken, and beef were the most available food items among the food groups classified in this study (See additional file 1). Lettuce, carrots, and tomatoes were also highly available among fresh vegetables; however, potatoes dominated this commodity [4]. Potato consumption was quite low among survey respondents, consumed by only 1.6% of the population. Potatoes were not included in the list of food items asked of the respondents because consumption of raw produce was of interest in this study and potatoes are rarely eaten raw. The reported consumption of potatoes likely reflects the consumption of cooked potatoes as opposed to raw potatoes and is most likely underreported as it represents open-ended responses given by respondents. In fact, a higher proportion of respondents reported consuming potato salad (8.2%) when asked specifically about potato salad consumption. The high prevalence of yogurt consumption is consistent with its availability, which has increased substantially in Canada over the past decade [4].

### Gender

The consumption of certain food items varied significantly by gender. Food items that were more likely to be consumed by males included meat products, unpasteurized juice and bean sprouts, whereas females were more likely to consume more fruits and vegetables, and dairy products. These consumption patterns are consistent with those found in a number of other dietary surveys [28-32]. Studies conducted in Great Britain also observed gender differences in food preferences, reporting that women consumed more fruits and vegetables compared to men, and had a higher preference for cultured dairy products such as cottage cheese and yogurt [33,34].

The greater consumption of fruits and vegetables and yogurt by females compared to males, appear to be more healthful food choices, suggesting that females may choose to eat certain foods perceived to be healthy and avoid foods considered to be unhealthy (e.g. fat avoidance). The particularly high consumption of lemons by females (compared to males) (OR = 6.53, 95% CI = 1.62, 26.26) could be the result of adding lemon to water, which may be viewed as a healthful practice or taste preference by females.

Previous studies have shown that, in general, women engage in healthier eating patterns compared to men [35] and are more motivated to respond to health messages focused on diet [28]. Motivation to practice healthier diets among women may reflect the greater importance of self image and physical appearance to women compared to men [36,37]. Such motivating factors have been associated with healthier diets [38,39] and higher intakes of fruits and vegetables [40].

Along with the notion of healthier eating patterns among women than men, this study also found that males are significantly more likely to consume food items considered high risk for the transmission of certain enteric pathogens, such as unpasteurized juice, bean sprouts, and undercooked meat. This is consistent with previous studies that show that women are more concerned than men with food safety issues [41,42]. These findings highlight an opportunity for health and food safety education targeted at males to help increase their awareness of the health risk involved in the consumption and preparation of certain food items. More detailed information on the specific high-risk foods consumed by males in the target population would be useful to help make effective public health campaigns.

### Age

Significant differences in food consumption by age group were also observed in this study. In general, fruits, vegetables, eggs, cottage cheese, fish, and nuts were more likely to be consumed among elderly individuals than younger individuals, whereas children and adolescents were more likely to consume dairy products such as milk, ice cream, and cheese. Adults consumed intermediate amounts of most food items compared to the other age categories. However, adults had higher consumption of herbs, yogurt, cheese, tofu and steak. Previous studies confirm that food intake varies among age groups [30,43,44].

Specific differences found between age groups that corroborate our findings include greater consumption of milk and dairy products among children compared to older individuals [31,43,45], and increased intake of fruits and vegetables associated with increasing age [31,43,46,47]. The finding that older respondents were less likely to consume milk, especially chocolate milk, compared to younger respondents was anticipated since the Canada Food Guide recommends children under 17 years of age consume two to four servings of milk each day for sufficient calcium intake [48]. Perhaps the higher consumption of chocolate milk among children and adolescents suggests a preference for sweetened beverages, given that recent studies on beverage consumption among children suggest that changes in milk consumption may be the result of sugar-sweetened beverages displacing milk [49,50].

Although soymilk and tofu consumption was generally low among respondents, elderly individuals showed the highest consumption of soymilk, and tofu consumption was highest among individuals between 25 and 54 years of age. While information on current intake of soy is well known among Asian populations [51,52], where soy foods are considered a staple part of the diet, information is relatively scarce among Western populations. A European study evaluating soy product consumption found that soy dairy substitutes were the most frequently consumed soy foods, although soy product consumption was relatively low.

Significant differences were found between age categories and egg consumption. Elderly respondents were significantly more likely to consume eggs, including undercooked eggs, than other respondents. These results differ from other study findings [41,53], which reported that consuming undercooked eggs was less common among the elderly. The high proportion of elderly individuals consuming runny eggs implies that this age group may be unaware of the health risks associated with eating undercooked eggs. Raw or undercooked eggs have been identified as a major source of *Salmonella *Enteritidis and Typhimurium infections [54]. Thus, the higher consumption of undercooked eggs among older respondents is important from a public health perspective because salmonellosis is particularly severe for this age group [55,56]. Food safety messages could be targeted towards the elderly and include information on health risks associated with eating raw or undercooked eggs, while emphasizing the importance of cooking eggs well to prevent illness.

The consumption of chicken nuggets, hamburgers not made in the home, and deli meats were highest among children, adolescents, and young adults compared to older individuals. This suggests that respondents in the younger age categories, or perhaps their parents, may be more interested in convenience food and food prepared outside of the home. Studies on food consumption trends show comparable findings among adolescents and young adults, where the consumption of cheeseburgers, pizza, ham, and salami has increased [27,31] representative of inexpensive food usually consumed outside of the home. These findings may also serve to explain the general low fruit and vegetable consumption among the younger age groups compared to the elderly age group. Kearney, Hulshof and Gibney [57] reported that, with increasing age, there was a decrease in the proportion of eating any meals outside of the home, and younger individuals were more likely to have their lunchtime meal outside of the home. Thus, the higher consumption of deli meats among individuals younger than 12 years may be related to meals eaten at school, where lunches may often consist of deli meat sandwiches.

The results presented here highlight differences in food consumption likely due to taste and preference, but differences in dietary choice by age may also be attributed, in part, to cohort effects [43]. For example, the diet of older individuals may partially reflect their habits in earlier decades. In addition, a variety of health conditions can dictate changes in diet as people age. However, in this study, only a very small proportion (5%) of respondents followed a special diet for various medical conditions, of which 66% were over the age of 55 years.

### Household dietary patterns

On average, the number of meals reported as being prepared within the home (25.8 meals) within the past seven days was much higher than those meals reported as being prepared outside of the home (3.3 meals), at a restaurant location. In addition, the weekly average number of meals that consisted of leftover food items (2.4 meals) was just slightly below that of the average number of restaurant meals. However, it is unknown whether the leftover meals had originally been prepared within the home. Nevertheless, these observations suggest that households in the study area still prepare the majority of their meals at home.

The results presented here are comparable to those found in the Canadian Community Health Survey [11] on eating habits, which reported that the majority of patrons of fast-food establishments selected pizza and sandwiches as their preferred meal choice. On average, about half of grocery foods purchased were basic raw ingredients, while half of purchased food items consisted of ready-to-eat and ready-to-cook foods. In addition, the average time spent on evening meal preparation in the home was 44 minutes. These findings may be somewhat unexpected as current consumer trends reveal a decrease in the time spent in meal preparation [58,59] and increased demand, purchase, and consumption of ready prepared foods and fast-food meals in response to evolving lifestyle changes [58,60-66]. Therefore, despite emerging trends in the food industry, it appears that many households still allocate time for meal preparation.

### Study Limitations

This study had several limitations. This study focused on food consumption patterns from a food safety perspective, and consumption of raw food items was of particular interest. As a result, food consumption patterns reported by respondents may not be entirely reflective of their usual consumption patterns since some food items, such as vegetables that are usually consumed cooked (i.e. potatoes), were not included in the survey. Future surveys should incorporate questions on the consumption of both cooked and raw food items.

Data on food consumption were subdivided on single demographic variables: gender and age. Demographic characteristics are seldom statistically independent of one another. For example, education and income have been shown to be important determinants in food intake [28,43,64,65]. In addition, since food choices are the result of a complex mixture of interacting social, cultural, and other environmental factors [40,66-68], it is possible that the observed differences among gender and age may not be due to these factors alone. Nevertheless, assessing the overall effects of both gender and age provides insight into the food consumption practices in different gender and age groupings necessary to support outbreak investigations and risk assessments, and may prompt further analysis.

Consumption of some food items is likely to vary according to season, often based on availability and price [69,70]. This study only collected data during one season (winter), precluding analysis of seasonal variation. It was shown, however, that the majority of reported diets consumed by respondents in the past seven days were representative of a typical weekly diet. It is possible that seasonal variation in diet may not be large within Canada because of the availability of an extensive variety of foods throughout the four seasons. However, a British Columbia study found that many foods were seasonally consumed with higher consumption of fruits in the summer than in the winter [69]. Future surveys should attempt to account for seasonality in the survey design by implementing the survey in different seasons or over a calendar year [71].

Response bias due to factors such as recall, the use of proxy respondents, and social desirability may have affected the results. In an attempt to reduce recall bias, a list of food items was used for the food consumption questions to prompt the respondents' memory. In addition, respondents were given the opportunity to provide answers that may have been omitted in the food list. Although a shorter recall period, such as 24 hours, may have reduced recall bias compared to a seven-day recall, single-day intake data tend to over- or under-estimate the usual intake of an individual [72]. Multiple days of dietary intake data reduce the effects of day-to-day variability on estimates of usual food intakes [73] and we chose to use this method here.

Younger children, in particular, are less able to recall their dietary intake than adults [74], and a surrogate respondent is often required to obtain such information. Although proxy respondents may not know exactly what a child ate when they were not together, studies comparing reports of subjects and their proxy respondents indicate that proxy information is useful despite possible under-reporting [75,76]. In this study we attempted to minimize proxy response bias by asking proxy respondents who were most acquainted with the child's daily activities to provide information on the child's diet.

Finally, the accuracy of the recall method also depends on the honesty of the respondents. Studies have shown that social desirability, the tendency to overestimate desirable behaviours and underestimate undesirable ones [77], biases self-reports of food and nutritional intake [78-80]. Barros, Moreira and Oliveira [80] showed that respondents tended to over-report consumption of foods perceived to be healthy, such as vegetables, and under-report consumption of foods perceived to be unhealthy, such as white bread and beer. Methods to measure and control for socially desirable responses are needed to reduce potential bias in self-reported dietary intake.

Although the overall response rate in this survey was low, it was consistent with response rates found in other dietary surveys in Canada [81,82]. A low response rate suggests the potential for selection bias, potentially limiting external validity of the study if non-respondents were different from survey respondents [77]. Since we did not collect information on non-respondents, the extent of potential bias could only be made by comparing the survey respondents to the total Waterloo Region Census population. The differences between the survey respondents and the study population were expected and were most likely the result of the sampling method used. In addition, the sampling frame used was a list of residential telephone numbers and the survey was administered in English only. However, of the 7,142 eligible respondents, only 179 (2%) did not participate due to language or hearing problems. Future studies may consider a sampling strategy to include potential respondents who do not speak English, who do not have telephones, as well as those with unlisted telephone numbers and mobile phone users.

## Conclusion

This study established baseline data on actual food intake of individuals in a Canadian based population from a food safety perspective. Differences in food consumption patterns were influenced by gender and age, although other important socio-demographic variables may also explain differences in dietary intake. These data demonstrate that certain gender and age groups may have unbalanced diets, which may be cause for concern from a nutritional perspective. Food safety education could be combined with strategies to promote healthier diets with appropriate nutritional guidance. This information is useful for food safety risk assessments and provides a baseline against which future outbreak investigations can compare proportions reported from case-series of infected individuals. Of concern is the consumption of specific foods among certain gender and age groups, suggesting that there may be a need not only to conduct food safety education among all consumers, but to target specific demographic groups that may be at a higher risk of foodborne disease. The majority of households reported preparing and consuming most meals at home. This suggests that consumer awareness of, and responsibility for, proper hygiene and food safety practices in the home is essential.

## Competing interests

The authors declare that they have no competing interests.

## Authors' contributions

SM was the project facilitator and secured project funding. SM, RF, AN participated in the study concept and design. AN was responsible for data analysis, data interpretation and manuscript preparation. All authors provided input into the survey tool, and provided expert advice on data interpretation and discussion of the paper. All authors have approved the final version of the manuscript.

## Pre-publication history

The pre-publication history for this paper can be accessed here:



## Supplementary Material

Additional file 1 **Total proportion of food items consumed by survey respondents.** Click here for file

Additional file 2 **Odds ratio of reported consumption of food items by age group.** Click here for file
