# Supplementary material for: Food consumption patterns in the Waterloo Region, Ontario, Canada: a cross-sectional telephone survey
Source: BMC Public Health. 2008 Oct 24;8:370. doi: 10.1186/1471-2458-8-370 (PMC2585092; doi:10.1186/1471-2458-8-370)
Supplement: Additional file 1 — Total proportion of food items consumed by survey respondents. [file 1471-2458-8-370-S1.doc]

**Additional File 1**

Weighted percentage and confidence intervals of respondents consuming specific food items in the past seven days, Waterloo Region, Ontario, Canada, November 2005 - March 2006
