# Supplementary material for: Food consumption patterns in the Waterloo Region, Ontario, Canada: a cross-sectional telephone survey
Source: BMC Public Health. 2008 Oct 24;8:370. doi: 10.1186/1471-2458-8-370 (PMC2585092; doi:10.1186/1471-2458-8-370)
Supplement: Additional file 2 — Odds ratio of reported consumption of food items by age group. [file 1471-2458-8-370-S2.doc]

**Additional File 2**

Odds ratios of reported consumption of food items by age group (adjusted by gender), showing only those food items whose differences were statistically significant (*P*≤0.05) from the referent group (individuals ≥65 years of age), in the Waterloo Region, Ontario, Canada, November 2005 - March 2006
